# Achieving MDG 4 in Sub-Saharan Africa: What Has Contributed to the Accelerated Child Mortality Decline in Ghana?

**DOI:** 10.1371/journal.pone.0017774

**Published:** 2011-03-21

**Authors:** Haruyo Nakamura, Nayu Ikeda, Andrew Stickley, Rintaro Mori, Kenji Shibuya

**Affiliations:** 1 Japan International Cooperation Agency (JICA), Tokyo, Japan; 2 Department of Global Health Policy, Graduate School of Medicine, University of Tokyo, Tokyo, Japan; 3 London School of Hygiene and Tropical Medicine, London, United Kingdom; Indiana University, United States of America

## Abstract

**Background:**

Recent analyses have suggested an accelerated decline in child mortality in Ghana since 2000. This study examines the long-term child mortality trends in the country, relates them to changes in the key drivers of mortality decline, and assesses the feasibility of the country's MDG 4 attainment.

**Methodology:**

Data from five Demographic and Health Surveys (DHS) between 1988 and 2008 and the Maternal Health Survey 2007 were used to generate two-year estimates of under-five mortality rates back to 1967. Lowess regression fitted past and future trends towards 2015. A modified Poisson approach was applied on the person-period data created from the DHS 2003 and 2008 to examine determinants of under-five mortality and their contributions to the change in mortality. A policy-modelling system assessed the feasibility of the country's MDG 4 attainment.

**Findings:**

The under-five mortality rate has steadily declined over the past 40 years with acceleration since 2000, and is projected to reach between 45 and 69 per 1000 live births in 2015. Preceding birth interval (reference: 36+ months, relative risk [RR] increased as the interval shortened), bed net use (RR 0.71, 95% confidence interval [CI]: 0.52–0.95), maternal education (reference: secondary/higher, RR 1.71, 95% CI: 1.18–2.47 for primary), and maternal age at birth (reference: 17+ years, RR 2.13, 95% CI: 1.12–4.05) were primarily associated with under-five mortality. Increased bed-net use made a substantial contribution to the mortality decline. The scale-up of key interventions will allow the possibility of Ghana's MDG 4 attainment.

**Conclusions:**

National and global efforts for scaling up key child survival interventions in Ghana are paying off ― these concerted efforts need to be sustained in order to achieve MDG 4.

## Introduction

Millennium Development Goal 4 (MDG 4) is targeted at reducing global under-five mortality rates by two-thirds between 1990 and 2015.[1] The number of children dying before reaching the age of five decreased from over 12.6 million in 1990 to around 9 million in 2007 worldwide, with the global under-five mortality rate declining from 93 to 67 per 1,000 live births in this period.[2], [3], [4] However, it was reported that little or no progress has been made in 68 countdown priority countries, which are responsible for 97% of maternal and child deaths worldwide.[1] These priority countries are concentrated in sub-Saharan Africa, where the absolute number of under-five deaths increased from 4.2 to 4.6 million during the above-mentioned period, primarily due to the persistently high fertility levels as well as the continuing high rates of mortality.[4]

In Ghana, the MDG 4 target of a two-thirds reduction in under-five mortality equates to 40 deaths per 1,000 live births, relative to the figure in 1990 which was 120 per 1,000 live births.[2], [3] However, by 2007 the figure had only declined to 115.[3] The MDG Countdown Report in 2008 rated Ghana as a “No Progress” country and warned that the country had to reduce its under-five mortality rate by 12% annually on average between 2007 and 2015 in order to reach the MDG 4 target.[1]

In contrast, two recent surveys―the Maternal Health Survey (MHS) 2007 and the Demographic and Health Survey (DHS) 2008―have both suggested a high rate of decline since 2003 (26% and 28%, respectively).[1] A more recent systematic review of child mortality worldwide and the updated MDG Countdown Report 2010 have also indicated an accelerated decline since 2000.[5], [6] However, no study has explained this occurrence in detail.[6]

This study, therefore, attempts to estimate the most up-to-date under-five mortality trends in Ghana, relates them to changes in the key drivers of child mortality decline, and assesses the feasibility of the country's MDG 4 attainment given the current child mortality reduction efforts in place.

## Materials and Methods

### Trends in under-five mortality between 1967 and 2008

We used data from the five DHS conducted in Ghana between 1988 and 2008, and the MHS 2007. These were nationally representative cluster sample surveys that covered 4,406, 5,822, 6,003, 6,251, 10,858, and 11,778 households in 1988, 1993, 1998, 2003, 2007, and 2008, respectively.[7], [8] This cross-sectional survey data provided direct estimates of child mortality, as well as detailed information on household demographics, asset ownership, the health and nutritional status of women and children, the coverage of health care services, and current knowledge and practices related to health. Survey data were obtained from interviews undertaken by trained personnel.[9]

Mortality among children aged younger than five years was estimated for every two-year period before each respective survey going back to 1967, using the direct method based on the complete birth histories of women aged between 15 and 49 years. Periods of exposure and deaths were grouped into two calendar years, and a separate life table was constructed for each period in the birth histories to show the probability that children would die before their next birthday. We then calculated the individual death probability by counting the number of children born in a certain period of time and the number of children dying in the same period. Then we used the synthetic cohort life table approach, in which the probabilities of death for small age segments based on real cohort mortality experience were combined into the more common age segments.[10] We used biannual mortality estimates up to 22 years back from the respective survey year to minimize maternal recall bias. In total, 66 mortality estimates were generated for the period between 1967 and 2008.

Mortality trends from 1967 to 2008 were estimated by fitting Lowess regression of the natural log of mortality in children younger than five years to time with bandwidths ranging from 0.3 (representing high sensitivity to recent data) to 2.0 (lower sensitivity), while the trend towards 2015 was forecast with the same bandwidths. Mean annual reduction rates were calculated by using the results from the Lowess regression analysis with the 0.3 bandwidth, which can precisely detect changes that occur over time.[11]

### Determinants of under-five mortality

In order to examine potential determinants of under-five mortality in Ghana, we pooled data of children's records from DHS 2003 and 2008. We used only these two surveys as they allowed us to maximize the number of intervention variables that could be examined. The pooled data had 6,609 under-five children born to 4,259 ever-married women aged 15–49 years at the time of the survey. We then transformed individual records of children to survival-time data, in order to consider the time to event and censoring and reflect the changing rates of mortality during childhood. In this risk dataset, children first became at risk and came under observation at date of birth. A row represented each child for each age band in which he/she survived and was therefore at risk. The analysis-time variable was time since birth measured in months, and we split childhood into five age bands: the first month of life (age 0 months), months 1–5, months 6–11, months 12–23 and months 24–59. We obtained 27,011 episodes for 6,609 children. The total exposure time was 184,307 child-months and 499 children died. The median time of exiting the risk set due to experiencing the event or censoring was 26 months.

We employed Poisson regression with robust error variance to model a binary outcome of death.[12], [13], [14] The Poisson regression model is appropriate for analyzing the risk of rare events such as under-five mortality which is not very common. However, the error for estimated relative risks may be overestimated when Poisson regression is applied to binomial data. We therefore used a sandwich estimator to obtain robust variance estimates. We also accounted for the clustering of children by mothers and included fixed effects of survey years in the model, specifying the exposure time for each child within each age band as defined above. Our log-linear hazard function of an underlying risk of death was
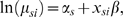
where *μ_si_* and *x_s__i_* were the risk of dying and a set of covariates, respectively, during age band *s* for child *i*; the exponential of the constant term, *α_s_*, was the vector of underlying age-specific risks of death given survival to the beginning of each age interval; and *β* was a set of estimated coefficients.

When selecting covariates for the model, we considered their relevance to child mortality in Ghana and potential confounding factors. Covariates consequently covered socioeconomic and demographic characteristics (urban/rural residence, region, religion, economic status, maternal age at birth, maternal highest education, maternal marital status, child's sex and multiplicity in birth) and maternal and child health practices (birth order, preceding birth interval, breastfeeding duration, use of a bed net, use of oral rehydration salt [ORS] and the number of antenatal care [ANC] visits). As a measure of economic status, we constructed wealth quintiles to make them comparable between the two surveys. We appended household survey datasets to estimate wealth index scores through a principal component analysis on the ownership of household assets that were available from both surveys.[15] Then, we computed cutoffs of wealth quintiles among household members in the pooled population. The wealth quintiles variable therefore refers to the economic level of a household to which the woman aged 15 to 49 years belongs. Furthermore, two dummy variables were created for the duration of breastfeeding to indicate for each age band whether children were breastfed for a sufficient length of time and whether breastfeeding terminated in preceding age bands or was still ongoing at the start of each age band: (1) children in age bands of 1–5 months or later who were never breastfed or breastfed for less than one month, and (2) children in age bands of 6–11 months or later who were never breastfed or had stopped being breastfed before entering the age band of 6–11 months. We determined cutoffs for the continuous variables of maternal age at birth and preceding birth interval to indicate early childbearing and short intervals, respectively, so that we could maintain a sufficient sample size for analysis and maximize the statistical significance of the model, while retaining consistency with the original purpose of the variable. After excluding children with missing values on any of these covariates, 24,228 episodes of 5,886 under-five children (3,359 in 2003 and 2,527 in 2008) were included in this regression.

For the relative contribution of each covariate to the changes in child mortality from 2003 to 2008, first we used the estimated beta coefficients and means of the explanatory variables in 2003 to calculate the risk of mortality in that year. Then, for each explanatory variable, we changed its values to its mean in 2008 to compute the change in the risk of mortality, setting other variables to their means in 2003. We divided the difference by the risk of mortality in 2003 to obtain the relative contribution of each explanatory variable.

### Potential impact of scaling up child-survival interventions

To estimate reductions in under-five mortality rates as a result of scaling up child-survival interventions in Ghana, we used the Lives Saved Tool (LiST) in the Spectrum Policy Modelling System.[16], [17] LiST calculates the impact of scaling up interventions on child death under the assumption that the number or proportion of deaths averted increases linearly as coverage increases from 0 to 100%, with some ability to incorporate a non-linear impact of component interventions, such as herd-immunity and increased quality. This programme assigns weights to the impact of each intervention to obtain the percentage of deaths averted across interventions.

LiST requires background information for projections of the future based on UN population division estimates and under-five mortality trends from the UN child mortality coordination group, child morbidity and nutritional status, deaths by causes, and coverage of child health interventions as well as assumptions concerning the efficacy of those interventions. The programme provides default values for the required information based on a review of scientific studies[17] and we used the built-in data, which included the DHS datasets, for the analysis of intervention impact on child mortality. The intervention coverage values come from the latest data sources, including DHS, Multiple Indicator Cluster Surveys, and other domestic household surveys conducted in the country. The particular coverage values for vaccinations and vitamin A came from UNICEF. The coverage values for 2015 were based on an expert review of trials on the effectiveness and consequent theoretical scale-up for each intervention. We set 2003 as the baseline year for our projection because the default data for the new LiST projections were collected to respond closely to the year 2003.

The tool projected the under-five mortality rates in 2009 and 2015 based on the background information explained above, and made an alternative child survival projection for 2015, in which the selected eight interventions were further up-scaled in pursuit of the MDG 4 target. The interventions were selected based on the causes of death among children aged under-five in Ghana (malaria, 32%; neonatal death, 29%; pneumonia, 15%; diarrhoea, 12%; HIV/AIDS, 6%; measles, 3%; and injuries, 3%)[1], the effectiveness of the interventions for these causes of death, and the past trends in intervention coverage in the country. Insecticide-treated materials are a 55% effective intervention to prevent malaria. Vitamin A as a preventive measure is 32% effective for diarrhoea and 19% effective for measles. Measles vaccination is 85% effective. Hib vaccination is 18% and DPT is 10% effective for pneumonia prevention. ORS is 93% and zinc is 23% effective for diarrhoea treatment.[16] Case management of neonatal severe infection was selected based on the evidence that coverage of postnatal care is closely related to institutional delivery and that specific case management can be provided by trained community workers, which suggests cost-effectiveness and swiftness of the intervention.[18]

The trend and determinant analyses were conducted using Stata/SE version 10.0 (StataCorp LP, College Station, TX, United States of America). LiST was used in the Spectrum version 3.45, downloaded from the website (Futures Institute, Glastonbury, CT, United States of America). The complex nature of the survey design, including such elements as stratification and clustering, was considered in the trend analysis and descriptive statistics of the study population.

## Results

### Trends in under-five mortality rates

**Figure 1:**
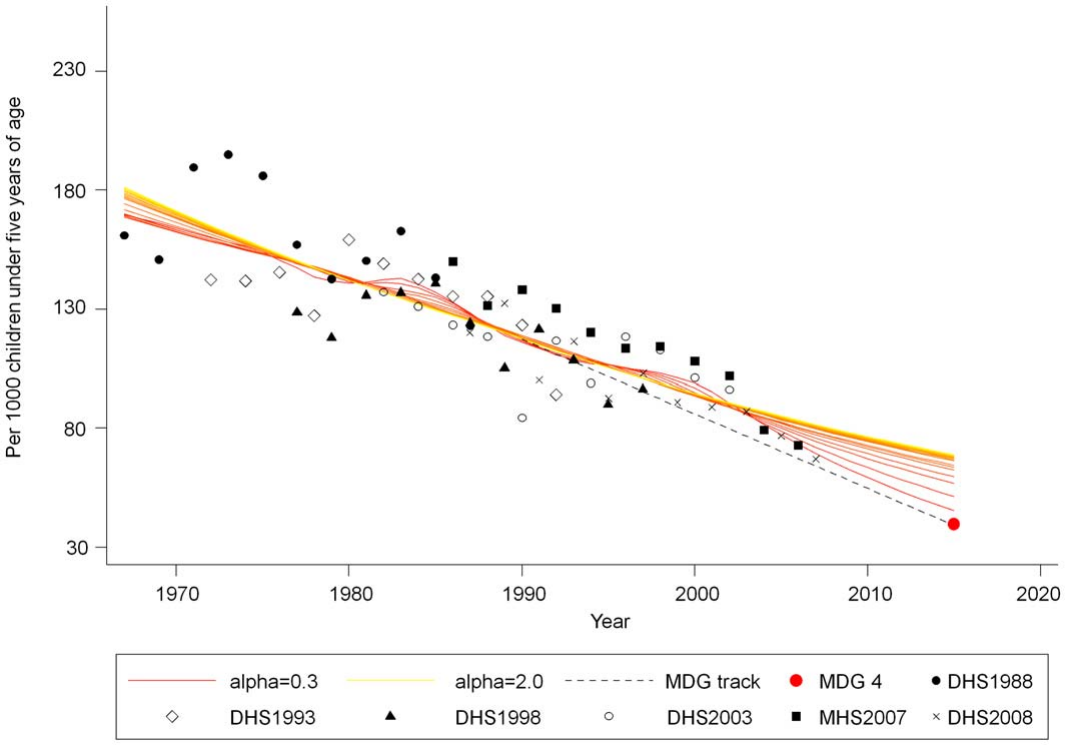
Two-year estimates of under-five mortality between 1967 and 2008 and projection towards 2015 in Ghana. Data are from Ghana Demographic and Health Surveys 1988–2008 and Ghana Maternal Health Survey 2007. Note: The MDG 4 target is 40 per 1000 live births, indicated as the red round-shaped point at the end of the dotted MDG track. MDG, Millennium Development Goal; DHS, Demographic and Health Survey; MHS, Maternal and Health Survey.

Figure 1 presents biannual trends in under-five mortality rates. Estimates from the Lowess regression indicated that under-five mortality had declined from 88 per 1,000 live births (95% CI: 86−89) in 2003 to 73 (95%CI: 66−80) in 2008. The under-five mortality rate steadily declined between 1967 and 2008 in Ghana and is projected to reach somewhere between 45 and 69 deaths per 1,000 live births in 2015. Mean annual rates of reduction in under-five mortality remained around 1.5–1.7% from the 1960s to the 1990s, but they have accelerated to 4.6% since 2000.[5]

### Factors associated with under-five mortality

Table S1 presents information on the socioeconomic, geographical and demographic characteristics and health service utilization of the respondents and their children aged younger than five years in the DHS 2003 and 2008. Between the two survey years, the poorest quintile and the poorer quintile decreased by 27% and 22%, while the richer quintile and the richest quintile increased by 50% and 54%, respectively. The proportion of women who had no education was reduced by 18% during the period. Coverage of bed net use for under-five children grew substantially by 196%, while the use of ORS and the coverage of ANC occurring more than 4 times also increased by 51% and 13%, respectively.

Table S2 shows the relative risk for under-five death in Ghana. Early child-bearing (<17 years) doubled the risk of under-five death (p = 0.021). Compared with children whose mothers had a secondary or higher education, children whose mothers received a primary education had a 1.7 times higher risk of dying before their fifth birthday (p = 0.004), while the likelihood also increased but was not statistically significant for those born to mothers who had no education (p = 0.103). A child who was born less than 1.5 years after a previous birth was 3.6 times more likely to die before their fifth birthday, compared to a baby born after a preceding birth interval of more than three years (p<0.001). Moreover, the risk of dying gradually reduced as the birth interval widened (RR 2.3, p<0·001 for 19–23 months; RR 1.6, p = 0.003 for 24–35 months). The risk of an under-five death increased three-fold when a child older than a month was never breastfed or breastfed for less than one month (p = 0.001), and the risk increases five-fold when a child older than six-months was never breastfed or breastfed for less than 6 months (p<0.001). The risk of child death was reduced by nearly 30% when all or some of the children aged younger than five slept under bed nets on the night before the interview (p = 0.023).

**Figure 2:**
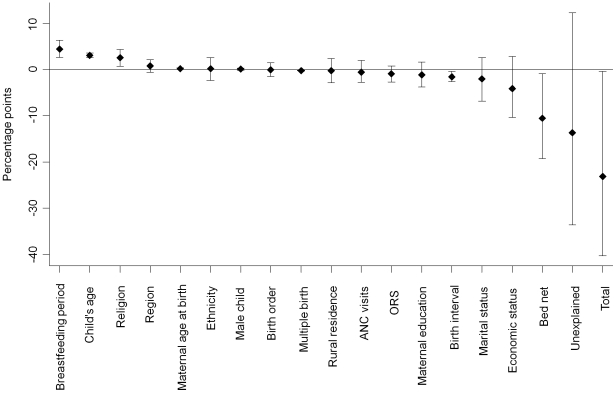
Estimated contributions of explanatory variables to the change in under-five mortality in Ghana between 2003 and 2008. Data are from Ghana Demographic and Health Surveys 2003 and 2008. The change in mean predicted probabilities of death under five years of age between 2003 and 2008 was decomposed into contributory factors.

Figure 2 presents information on how much each determinant has contributed to the mortality decline between 2003 and 2008. Out of 23.1 percentage points reduction in the probability of under-five mortality between the two consecutive survey years, −10.6 percentage points (95% CI: −19.2 to −1.0), came from an increased use of bed nets. The other significant factor was a widened birth interval, which contributed −1.6 percentage points (95% CI: −2.6 to −0.4) to the reduction in the probability of a child's death. Finally, −13.7 percentage points remained unexplained (95% CI: −33.6 to 12.2), which would include unobserved variables in the household surveys including immunization, and the use of zinc and antibiotics.[19]

### Mortality impact of changes in the intervention coverage

Table S3 presents the adjusted and projected coverage of the child survival interventions in 2003, 2009 and 2015 in Ghana. Using LiST the under-five mortality rate is estimated to be 68 per 1000 live births in 2009, which is within the range of the Lowess regression estimates of between 62 and 78 per 1000 live births. The intervention coverage was then projected to increase towards 2015, yielding an under-five mortality rate of 49 per 1000 live births, which was also within the Lowess estimates of 45 to 69 per 1000 live births.

We suggested an alternative scenario with the further up-scaling of eight interventions by 2015, which is indicated as “2015a” in the fourth column of Table S3. If these interventions were up-scaled to this alternative level and other interventions remained at the 2015 level, Ghana would attain its MDG 4 target: 40 deaths per 1000 live births. In this scenario, Ghana can upscale the eight interventions through the following four strategies. First, extra efforts are made to further extend the target coverage of rapidly up-scaled insecticide-treated materials from 80% to 90%. Second, the successful three child-survival immunizations: i.e. measles, diphtheria, pertussis, tetanus (DPT), and Haemophilus influenzae type B (Hib), are provided altogether with vitamin A supplements, which could bring a reduction of up to 25% in under-five mortality in sub-Saharan Africa.[20], [21] Third, based on the assumption that 79.4% of women would have institutional delivery by 2015 in Ghana,[17] deaths caused by severe neonatal infections could be widely averted by the proper training of health personnel to continually monitor mothers before, during and after delivery and swiftly diagnose and treat neonatal illnesses, as coverage of postnatal care is closely related to institutional delivery. Finally, the use of ORS for children with diarrhoea is further promoted based on the fact that more than 90% of mothers have knowledge of ORS,[22] and use of zinc can be introduced as an adjunct to ORS.

## Discussion

We confirm a steady decline in under-five mortality between 1967 and 2008 with an accelerated decline since 2000. The mean annual rate of reduction: 4.6% since 2000 exceeded the rate required for achieving MDG4 (i.e. 4. 4%).[5] Our estimate is higher than the MDG 4 target, but is substantially lower than previous estimates[1] and consistent with new estimates,[5], [6] [23] which included the recent data from MHS and DHS.[8], [22]

This study analysed the trends and determininants of under-five mortality and the relative contribution of different factors to the decline in child mortality. This series of population-level analyses, together with the practical estimates of child survival intervention coverage as a supplementary tool, has suggested a way of directing policy-initiatives towards MDG 4 attainment, especially for other priority countries.

Specifically, we have identified bed net use for children aged under five and preceding birth interval as being important determinants of child mortality as well as major contributors to the decline in mortality. We also found that mothers' educational attainment, mother's age at birth and breatfeeding duration were important determinants of child mortality. The results of this study suggest that under-five mortality was successfully reduced by a combination of the recent scale up of child survival interventions and an overall improvement in the socioeconomic conditions in Ghana.

In addition to the factors identified in this study, there are several other possible reasons for the decline in child mortality in Ghana. First, Ghana's economic improvement must have been behind the success. In fact, Ghana has enjoyed constant increases in gross domestic product (GDP) over the past 15 years [24] and real GDP growth rates of 5 to 7 percent since 2003.[25] Second, the country's pursuit of MDG 4 attainment itself may have played a role and the scaling up of other key child survival interventions has certainly reduced under-five mortality. [22], [26] In particular, the coverage of the key child immunizations was successfully increased from less than 20% in 1980 to over 90% in 2007.[27] With a few exceptions, child-survival interventions were up-scaled by 10 to 30% in Ghana between 2003 and 2008.[22], [28]

Ghana has also achieved equity in intervention coverage. A recent analysis revealed that Ghana had the highest coverage in the richest quintile and the second poorest quintile among the 16 Sub-Saharan African Countries with 2009 estimated GDP per capita of less than International $1,000. [29] Various trials have shown remarkable reductions in child mortality with the case management of ill children by community health workers [30] and it can be conjectured that the piloting and scale-up of the nation-wide “High Impact Rapid Delivery (HIRD)” strategy,[31] together with a community health service delivery framework, Community-based Health Planning and Services (CHPS), may have played a pivotal role in accelerating child mortality reduction in Ghana, though a recent retrospective evaluation of HIRD highlighted the mixed results of the Program in West Africa.[32] Finally, the decentralization of the health system, initiated in 1996 by introducing sector-wide capitation grants and giving districts sufficient financial resources, has also been relatively successful in Ghana, owing to the early establishment of the District Health Management Team as a primary health care strategy in the 1980s.[33]

Several limitations of this study should be mentioned. First, the limited number of variables in the DHS datasets did not allow us to explore a wider range of intervention variables, particularly the direct impact of child survival interventions on under-five mortality, including immunizations and the case management of malaria, pneumonia and diarrhoea, as well as neonatal mortality, which accounts for 29% of under-five mortality in Ghana.[1] The limited nature of the variables examined in this study highlights the large unexplained element in the mortality reduction. In order to maximize the range of intervention variables, we used only DHS 2003 and 2008, which in turn reduced the number of observations in the analysis. Second, the nature of the cross-sectional data also did not allow us to investigate any causal relations between variables or effectiveness of interventions on child mortality. Third, DHS-style direct birth histories may be subject to recall bias and/or the underreporting of child mortality by mothers, which could result in more than a 7% underestimation of deaths.[23], [34] The non-inclusion of other data sets meant that the current study may also have been subject to this potential problem. Finally, the LiST assumption of a linear decrease in the number of deaths with increasing intervention coverage remains somewhat uncertain as there are non-linear effects on child mortality which are not controlled by the tool.

In conclusion, Ghana has experienced a slow, but nevertheless, steady decline in its under-five mortality rate over the past forty years with an accelerated reduction since 2000. National and global efforts for scaling up key child survival interventions in Ghana are paying off and need to be sustained. Strategic scale-up of key interventions will lead the country even further towards the attainment of its MDG 4 target. Identification and enhancement of key child-survival interventions, following a thorough examination of each country's specific context, are recommended for other low-income, “No progress” countries, particularly in sub-Saharan Africa.

## Supporting Information

Table S1 **Socioeconomic, geographic and demographic characteristics and health service utilization of women aged 15 to 49 years and their children aged under five, the Demographic and Health Surveys 2003 and 2008, Ghana.** Values are percentages with 95% confidence intervals in parentheses.
(DOCX)

Table S2 **Relative risk of death among children aged under five years of age in Ghana, the Demographic and Health Surveys 2003 and 2008, Ghana.** CI, confidence interval; RR, relative risk.
(DOCX)

Table S3 **Projected/alternative coverage (%) of key child-survival interventions in Ghana.** BCG, Bacillus Calmette-Guérin; DPT, diphtheria, pertussis, tetanus; Hib, Haemophilus influenzae type B.
(DOCX)
