# Supplementary material for: Achieving MDG 4 in Sub-Saharan Africa: What Has Contributed to the Accelerated Child Mortality Decline in Ghana?
Source: PLoS One. 2011 Mar 21;6(3):e17774. doi: 10.1371/journal.pone.0017774 (PMC3061869; doi:10.1371/journal.pone.0017774)
Supplement: Table S1 — Socioeconomic, geographic and demographic characteristics and health service utilization of women aged 15 to 49 years and their children aged under five, the Demographic and Health Surveys 2003 and 2008, Ghana. Values are percentages with 95% confidence intervals in parentheses. (DOCX) [file pone.0017774.s001.docx]

**Table S1. Socioeconomic, geographic and demographic characteristics and health service utilization of women aged 15 to 49 years and their children aged under five, the Demographic and Health Surveys 2003 and 2008, Ghana.**

| Characteristics | | 2003 (N=3,747) | | 2008 (N=2,862)) | |
| --- | --- | --- | --- | --- | --- |
| Type of Residence | |  |  |  |  |
|  | Urban | 32.5 | (30.0–35.2) | 37.1 | (34.3–39.9) |
|  | Rural | 67.5 | (64.8–70.1) | 62.9 | (60.1–65.7) |
| Region | |  |  |  |  |
|  | Greater Accra | 10.4 | (9.0–12.1) | 11.8 | (10.1–13.8) |
|  | Western | 10.1 | (8.5–11.9) | 9.3 | (7.8–11.0) |
|  | Central | 8.6 | (6.8–10.6) | 9.5 | (8.0–11.4) |
|  | Volta | 8.1 | (6.9–9.5) | 8.8 | (7.2–10.7) |
|  | Eastern | 9.9 | (8.5–11.4) | 8.2 | (7.1–9.4) |
|  | Ashanti | 18.5 | (16.2–21.0) | 18.9 | (16.5–21.5) |
|  | Brong Ahafo | 11.1 | (9.6–12.8) | 9.4 | (8.1–10.8) |
|  | Upper West, Eastern & Northern | 23.4 | (21.1–25.9) | 24.2 | (21.6–27.0) |
| Religion | |  |  |  |  |
|  | No religion/other | 6.7 | (5.4–8.3) | 4.6 | (3.5–6.1) |
|  | Roman Catholic | 12.0 | (10.3–13.9) | 11.9 | (10.1–14) |
|  | Protestant | 14.5 | (12.6–16.7) | 12.3 | (10.6–14.4) |
|  | Other Christian | 44.2 | (41.2–47.2) | 45.4 | (42.2–48.6) |
|  | Moslem | 18.4 | (15.6–21.5) | 19.1 | (15.6–23.3) |
|  | Traditional/Spiritual | 4.3 | (3.2–5.7) | 6.6 | (4.6–9.3) |
| Ethnic | |  |  |  |  |
|  | Akan | 46.3 | (42.7–49.9) | 44.8 | (41.2–48.6) |
|  | Ga/Dangme | 7.2 | (5.5–9.4) | 5.0 | (3.8–6.7) |
|  | Ewe | 12.1 | (10.2–14.4) | 12.7 | (10.5–15.3) |
|  | Guan | 2.4 | (1.7–3.5) | 2.9 | (1.6–5.2) |
|  | Mole-Dagbani | 16.8 | (14.1–20.0) | 20.9 | (17.6–24.5) |
|  | Grussi | 2.9 | (1.8–4.5) | 2.9 | (2.0–4.1) |
|  | Gruma | 4.3 | (2.5–7.2) | 5.9 | (4.1–8.6) |
|  | Others | 8.0 | (5.9–10.8) | 4.9 | (3.5–6.7) |
| Wealth quintiles | |  |  |  |  |
|  | Poorest | 31.6 | (28.7–34.7) | 23.8 | (20.6–27.3) |
|  | Poorer | 28.2 | (25.6–31.0) | 21.0 | (18.4–23.9) |
|  | Average | 16.3 | (14.3–18.5) | 20.1 | (17.5–23.0) |
|  | Richer | 12.7 | (11.1–14.5) | 18.9 | (16.5–21.6) |
|  | Richest | 11.1 | (9.2–13.5) | 16.2 | (14.2–18.4) |
| Maternal highest education | |  |  |  |  |
|  | None | 41.2 | (38.2–44.1) | 34.0 | (31.2–37.0) |
|  | Primary | 22.8 | (20.8–25.0) | 24.9 | (22.5–27.4) |
|  | Secondary/higher | 36.0 | (33.3–38.8) | 41.1 | (38.1–44.2) |

Values are percentages with 95% confidence intervals in parentheses.

**Table S1. (Continued)**

| Characteristics | | 2003 (N=3,747) | | 2008 (N=2,862)) | |
| --- | --- | --- | --- | --- | --- |
| Maternal age at birth (years) | |  |  |  |  |
|  | 13–16 | 1.4 | (1.1–1.9) | 1.5 | (1.1–2.1) |
|  | 17–48 | 98.6 | (98.1–99.0) | 98.5 | (97.9–98.9) |
| Marital status | |  |  |  |  |
|  | Married | 83.5 | (81.3–85.4) | 74.6 | (71.9–77.1) |
|  | Living together | 9.5 | (7.7–11.6) | 19.6 | (17.3–22.1) |
|  | Widowed | 1.2 | (0.9–1.8) | 1.0 | (0.7–1.6) |
|  | Divorced | 2.0 | (1.4–2.7) | 2.0 | (1.3–3.0) |
|  | Not living together | 3.8 | (3.1–4.8) | 2.8 | (2.1–3.7) |
| ORS used | | 7.4 | (6.3–8.7) | 11.8 | (10.0–13.8) |
| Bed net used | | 17.1 | (14.9–19.6) | 49.2 | (46.0–52.4) |
| Having more than 4 ANC visits | | 69.1 | (66.1–71.9) | 77.9 | (75.2–80.3) |
| Child's sex | |  |  |  |  |
|  | Female | 51.0 | (49.3–52.8) | 52.2 | (50.1–54.3) |
|  | Male | 49.0 | (47.2–50.7) | 47.8 | (45.7–49.9) |
| Multiplicity in birth | | 4.0 | (3.1–5.3) | 4.6 | (3.5–6.0) |
| Preceding birth interval (months) | |  |  |  |  |
|  | First birth | 20.7 | (19.3–22.3) | 20.7 | (19.2–22.3) |
|  | 1–18 | 4.7 | (3.9–5.5) | 3.8 | (3.0–4.8) |
|  | 19–23 | 6.1 | (6.1–7.1) | 7.3 | (6.3–8.4) |
|  | 24–35 | 24.1 | (22.6–25.8) | 21.8 | (19.9–23.7) |
|  | 36+ | 44.4 | (42.4–46.3) | 46.5 | (44.4–48.6) |
| Breastfeeding duration | |  |  |  |  |
|  | <1 month for older than 1-month old | 0.7 | (0.4–1.4) | 1.5 | (1.0–2.3) |
|  | <6 months for older than 6-month old | 1.2 | (0.8–1.9) | 2.4 | (1.7–3.3) |

Values are percentages with 95% confidence intervals in parentheses.
