# Supplementary material for: Achieving MDG 4 in Sub-Saharan Africa: What Has Contributed to the Accelerated Child Mortality Decline in Ghana?
Source: PLoS One. 2011 Mar 21;6(3):e17774. doi: 10.1371/journal.pone.0017774 (PMC3061869; doi:10.1371/journal.pone.0017774)
Supplement: Table S2 — Relative risk of death among children aged under five years of age in Ghana, the Demographic and Health Surveys 2003 and 2008, Ghana. CI, confidence interval; RR, relative risk. (DOCX) [file pone.0017774.s002.docx]

**Table S2. Relative risk of death among children aged under five years of age in Ghana, the Demographic and Health Surveys 2003 and 2008, Ghana.**

| Variable | Category | RR | 95%CI | |
| --- | --- | --- | --- | --- |
| Type of residence | |  |  |  |
|  | Rural | 1.04 | 0.70 | 1.57 |
|  | Urban | 1.00 | Reference | |
| Region |  |  |  |  |
|  | Western | 0.78 | 0.41 | 1.49 |
|  | Central | 0.78 | 0.38 | 1.59 |
|  | Greater Accra | 1.00 | Reference | |
|  | Volta | 0.95 | 0.46 | 1.96 |
|  | Eastern | 0.76 | 0.41 | 1.43 |
|  | Ashanti | 0.85 | 0.49 | 1.49 |
|  | Brong Ahafo | 0.78 | 0.41 | 1.51 |
|  | Northern | 0.92 | 0.48 | 1.75 |
| Religion |  |  |  |  |
|  | No religion/other | 0.76 | 0.45 | 1.29 |
|  | Roman Catholic | 1.08 | 0.74 | 1.59 |
|  | Protestant | 0.56 | 0.32 | 1.00 |
|  | Other Christian | 0.91 | 0.63 | 1.33 |
|  | Moslem | 1.00 | Reference | |
|  | Traditional/Spiritual | 1.40 | 0.89 | 2.19 |
| Ethnic |  |  |  |  |
|  | Akan | 1.00 | Reference | |
|  | Ga/Dangme | 1.02 | 0.57 | 1.82 |
|  | Ewe | 0.72 | 0.44 | 1.17 |
|  | Guan | 1.30 | 0.66 | 2.57 |
|  | Mole-Dagbani | 0.86 | 0.55 | 1.35 |
|  | Grussi | 1.37 | 0.78 | 2.40 |
|  | Gruma | 1.09 | 0.67 | 1.79 |
|  | Others | 0.79 | 0.44 | 1.42 |
| Economic status | |  |  |  |
|  | Poorest | 1.13 | 0.58 | 2.19 |
|  | Poorer | 1.29 | 0.69 | 2.43 |
|  | Average | 1.04 | 0.56 | 1.95 |
|  | Richer | 0.81 | 0.45 | 1.45 |
|  | Richest | 1.00 | Reference | |
| Mother's educational achievement | |  |  |  |
|  | No education | 1.39 | 0.94 | 2.06 |
|  | Primary education | 1.71 | 1.18 | 2.47 |
|  | Secondary/higher | 1.00 | Reference | |
| Mother's age at birth (years) | |  |  |  |
|  | 13-16 | 2.13 | 1.12 | 4.05 |
|  | 17-48 | 1.00 | Reference | |

CI, confidence interval; RR, relative risk.

**Table S2. (Continued)**

| Variable | Category | RR | 95%CI | |
| --- | --- | --- | --- | --- |
| Mother's marital status | |  |  |  |
|  | Married | 1.00 | Reference | |
|  | Living together | 0.84 | 0.54 | 1.29 |
|  | Widowed | 1.08 | 0.48 | 2.45 |
|  | Divorced | 0.92 | 0.40 | 2.13 |
|  | Not living together | 1.05 | 0.52 | 2.09 |
| ORS |  |  |  |  |
|  | Not used | 1.00 | Reference | |
|  | Used | 0.79 | 0.52 | 1.20 |
| Bed net |  |  |  |  |
|  | Not used | 1.00 | Reference | |
|  | Used | 0.71 | 0.52 | 0.95 |
| ANC 4 visits or more | |  |  |  |
|  | Not visited | 1.00 | Reference | |
|  | Visited | 0.94 | 0.72 | 1.22 |
| Child's sex |  |  |  |  |
|  | Female | 1.00 | Reference | |
|  | Male | 0.95 | 0.75 | 1.20 |
| Multiplicity in birth | |  |  |  |
|  | Single | 1.00 | Reference | |
|  | Multiple | 5.47 | 3.84 | 7.80 |
| Birth order |  | 1.00 | 0.94 | 1.07 |
| Preceding birth interval (months) | |  |  |  |
|  | First birth | 1.80 | 1.20 | 2.68 |
|  | 1-18 | 3.63 | 2.33 | 5.67 |
|  | 19-23 | 2.26 | 1.48 | 3.45 |
|  | 24-35 | 1.58 | 1.16 | 2.13 |
|  | 36+ | 1.00 | Reference | |
| Breastfeeding duration | |  |  |  |
|  | <1 month for older than 1-month old | 4.17 | 1.79 | 9.70 |
|  | <6 months for older than 6-month old | 6.02 | 2.45 | 14.79 |
| Age interval constants | |  |  |  |
|  | 0 months | 0.02 | 0.01 | 0.03 |
|  | 1-5 months | 0.00 | 0.00 | 0.00 |
|  | 6-11 months | 0.00 | 0.00 | 0.00 |
|  | 12-23 months | 0.00 | 0.00 | 0.00 |
|  | 24-59 months | 0.00 | 0.00 | 0.00 |
| Survey year |  |  |  |  |
|  | 2003 | 1.00 | Reference | |
|  | 2008 | 0.86 | 0.66 | 1.13 |

ANC, antenatal care; CI, confidence interval; ORS, oral rehydration salt; RR, relative risk.
