# Supplementary material for: Achieving MDG 4 in Sub-Saharan Africa: What Has Contributed to the Accelerated Child Mortality Decline in Ghana?
Source: PLoS One. 2011 Mar 21;6(3):e17774. doi: 10.1371/journal.pone.0017774 (PMC3061869; doi:10.1371/journal.pone.0017774)
Supplement: Table S3 — Projected/alternative coverage (%) of key child-survival interventions in Ghana. BCG, Bacillus Calmette-Guérin; DPT, diphtheria, pertussis, tetanus; Hib, Haemophilus influenzae type B. (DOCX) [file pone.0017774.s003.docx]

**Table S3. Projected/ alternative coverage (%) of key child-survival interventions in Ghana.**

| Interventions | 2003 | | 2009 | 2015 | 2015a |
| --- | --- | --- | --- | --- | --- |
| Antenatal care | | 32.5 | 59.6 | 80.0 |  |
| Tetanus toxoid | | 89.0 | 65.2 | 90.0 |  |
| Institutional delivery | | 8.9 | 63.2 | 79.4 |  |
| Skilled birth attendance | | 13.1 | 64.4 | 80.0 |  |
| Kangaroo mother care | | 16.0 | 47.3 | 60.0 |  |
| Breastfeeding promotion | | 23.5 | 38.2 | 80.0 |  |
| Use of improved water source | | 96.2 | 84.0 | 84.0 |  |
| Use of water connection in the home | | 6.7 | 18.1 | 29.5 |  |
| Improved excreta disposal (latrine/ toilet) | | 23.5 | 17.5 | 29.5 |  |
| Hygienic disposal children's stools | | 22.5 | 31.4 | 54.2 |  |
| Insecticide-treated materials or indoor residual spraying | | 0.0 | 41.9 | 80.0 | 90.0 |
| Vitamin A for prevention | | 87.0 | 62.2 | 80.0 | 95.0 |
| Measles vaccination | | 76.0 | 90.1 | 90.1 | 95.0 |
| Hib vaccination | | 0.0 | 92.9 | 92.9 | 95.0 |
| DPT vaccination | | 81.0 | 89.1 | 90.0 | 95.0 |
| Polio vaccination | | 82.0 | 84.1 | 90.0 |  |
| BCG vaccination | | 95.0 | 93.7 | 93.7 |  |
| Case management of neonatal severe infection with full supportive care | | 3.0 | 8.8 | 25.0 | 75.0 |
| Oral rehydration salt | | 65.8 | 53.9 | 80.0 | 90.0 |
| Antibiotics for dysentery | | 16.0 | 30.5 | 62.9 |  |
| Zinc for treatment | | 0.0 | 33.9 | 66.3 | 80.0 |
| Case management of pneumonia (oral antibiotics) | | 21.5 | 38.2 | 73.0 |  |
| Antimalarials | | 0.0 | 52.8 | 80.0 |  |

BCG, Bacillus Calmette-Guérin; DPT, diphtheria, pertussis, tetanus; Hib, Haemophilus influenzae type B.
